# Synthesis of imidazol-1-yl-acetic acid hydrochloride: A key intermediate for zoledronic acid

**DOI:** 10.3762/bjoc.4.42

**Published:** 2008-11-17

**Authors:** Santosh Kumar Singh, Narendra Manne, Purna Chandra Ray, Manojit Pal

**Affiliations:** 1 New Drug Discovery, Matrix Laboratories Limited, Anrich Industrial Estate, Bollaram, Jinnaram Mandal, Medak District, Andhra Pradesh, India-502 325.

**Keywords:** ester, N-alkylation, imidazol-1-yl-acetic acid hydrochloride, zoledronic acid

## Abstract

A convenient and practical synthesis of imidazol-1-yl-acetic acid hydrochloride was achieved via N-alkylation of imidazole using *tert*-butyl chloroacetate followed by a non-aqueous ester cleavage of the resulting imidazol-1-yl-acetic acid *tert*-butyl ester in the presence of titanium tetrachloride. The synthesized imidazol-1-yl-acetic acid hydrochloride was then utilized to prepare zoledronic acid.

## Introduction

**Figure 1:**
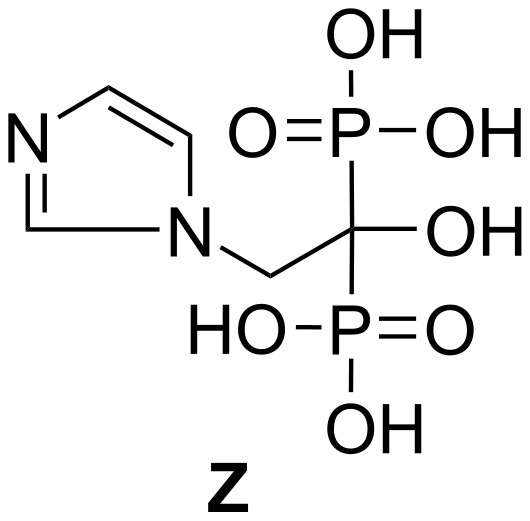
Structure of zoledronic acid.

Zoledronic acid (**Z**, Figure 1) [1–7] is a third-generation bisphosphonate (or diphosphonate) derivative characterized by a side chain that includes an imidazole ring. It inhibits osteoclast action and bone resorption and is used to treat tumor-induced hypercalcemia i.e. a disease condition characterized by the high levels of calcium in the blood (normal range 9–10.5 mg/dL or 2.2–2.6 mmol/L) usually caused by certain types of cancer. Zoledronic acid is also used along with the cancer chemotherapy to treat bone damage caused by multiple myeloma (a type of cancer of plasma cells that are part of the immune system cells in bone marrow and produce antibodies) or by cancer that began in another part of the body but has spread to the bones. While the use of zoledronic acid can neither suppress nor stop cancer spreading, it can be used to treat bone disease in patients who are suffering from cancer. It works by slowing bone breakdown and decreasing the amount of calcium released from the bones into the blood. It is commercially available in products sold under the brand name Zometa™ in vials as a sterile powder or solution for intravenous infusion. As part of our ongoing project under the new drug discovery program we had a requirement for compound **Z** in quantity. However, we encountered several difficulties while following the existing methods, the most critical one being the isolation of imidazol-1-yl-acetic acid, a key precursor of **Z**, from aqueous media (see later for a discussion). We then decided to develop a more straightforward and practical route to generate **Z**, also suitable for its scale-up. Herein we report our detailed study on the synthesis of **Z** following a much improved procedure.

## Results and Discussion

**Scheme 1:**
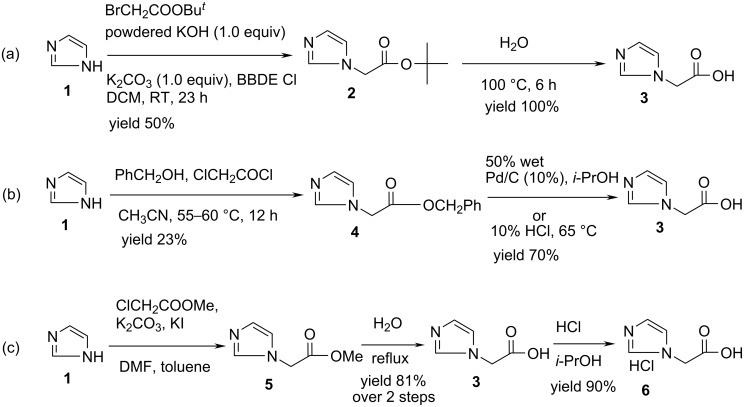
Reported methods for the synthesis of imidazol-1-yl-acetic acid (**3**) [8–10].

Various routes for the preparation of imidazol-1-yl-acetic acid (**3**), a key precursor of zoledronic acid (**Z**), have been reported [8–10]. Some of these are summarized in Scheme 1. Thus, imidazol-1-yl-acetic acid *tert*-butyl ester (**2**) was synthesized in 50% yield (Method a, Scheme 1) via the reaction of 2.0 equiv of imidazole (**1**) with 1.0 equiv of *tert*-butyl bromoacetate in the presence of an expensive catalyst e.g. bis-[2-(*N*-benzyl-*N*,*N*-diethylammonio)ethyl]ether dichloride (BBDE Cl) and a combination of bases such as KOH-K_2_CO_3_ followed by ester hydrolysis in water at 100 °C [10]. In another process [8] for the preparation of zoledronic acid, a benzyl ester of imidazole-1-yl-acetic acid **4** was prepared by reacting imidazole (**1**) with benzyl chloroacetate generated in situ from benzyl alcohol and chloroacetyl chloride. The ester **4** was then converted to compound **3** either by debenzylation using 10% Pd/C (50% wet) or by acidic hydrolysis using 10% hydrochloric acid (Method b, Scheme 1). The third process [9] involved the reaction of methyl chloroacetate with **1** in a mixture of solvents followed by hydrolysis to afford the compound **3** which was then converted to its hydrochloride salt **6** (Method c, Scheme 1).

It is now evident that all these processes (i) involved a complicated alkylation procedure as a first step, (ii) required the use of an aqueous media in the last step to produce the acid **3** and (iii) required an additional step to produce the hydrochloride salt thereby increasing the number of steps. Moreover, due to its high degree of water solubility isolation of compound **3** often involved evaporation of water instead of extraction of the aqueous solution by a common organic solvent. All these drawbacks prevented us utilizing any of these known processes for the preparation of **3** in large scale.

**Scheme 2:**
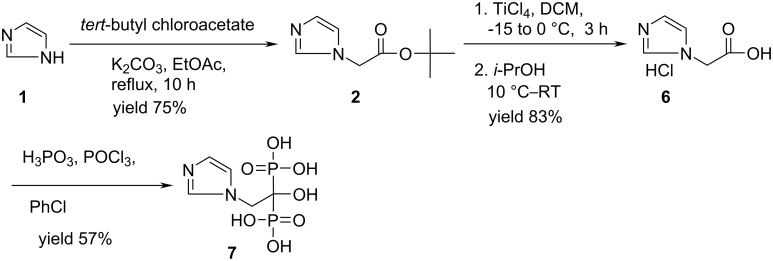
Preparation of imidazol-1-yl-acetic acid hydrochloride (**6**) and its conversion into zoledronic acid (**7**).

**Scheme 3:**
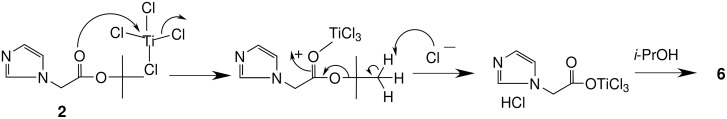
Suggested mechanism of TiCl_4_-mediated *tert*-butyl ester cleavage.

Our aim was to prepare compound **6** avoiding the difficulties mentioned above, particularly the aqueous hydrolysis of the corresponding ester precursor. Our synthesis of **6** is shown in Scheme 2. Based on the observations and results of earlier reports (step 1 of Method a, b & c, Scheme 1) we chose *tert*-butyl chloroacetate as an efficient and cheaper (compared to *tert*-butyl bromoacetate) alkylating agent for **1**. Accordingly, compound **2** was prepared by reacting **1** with stoichiometric amount of *tert*-butyl chloroacetate in the presence of K_2_CO_3_ in refluxing ethyl acetate. After usual work up the desired ester **2** was isolated in 75% yield. The ester **2** was then treated with TiCl_4_ in dichloromethane at low temperature to facilitate the non-aqueous cleavage of the *tert*-butyl ester moiety. Quenching the reaction mixture with *i*-PrOH provided the hydrochloride salt **6** as a crystalline solid in the same pot. This step is thus not only free from the use of aqueous media but also avoids aqueous work up followed by extraction with an organic solvent. Moreover, the hydrochloride salt **6** was isolated in 83% yield without using HCl. Determination of HCl-content indicated that the compound **6** contains ~ 1.0 mol of HCl along with 3.25% moisture. While the use of TiCl_4_ for the selective cleavage of *tert*-butyl ethers is a well known process [11] and has been studied for the cleavage of an ester in a cephalosporin derivative earlier [12], isolation of a hydrochloride salt product directly from the same reaction mixture is not common. A plausible mechanism for TiCl_4_ mediated cleavage of *tert*-butyl ester followed by salt formation in tandem is shown in Scheme 3. It is expected that interaction of the ester oxygen with TiCl_4_ facilitates the cleavage of the C–O bond attached to the *tert*-butyl group. The HCl generated during this conversion is trapped by the imidazole (to form a salt). Treatment with *i*-PrOH produces the desired acid **6**.

Having prepared the key intermediate **6**, it was converted to zoledronic acid (**7**) in 57% yield by reacting with H_3_PO_3_ and POCl_3_ according to the procedure shown in Scheme 2.

## Conclusion

In conclusion, we have reported a practical two-step direct synthesis of imidazol-1-yl-acetic acid hydrochloride, a key intermediate for the synthesis of zoledronic acid, in more than 5 g scale. Unlike earlier routes, the present process is free from the use of aqueous hydrolysis step thereby evaporation of water, and environmentally harmful hydrochloric acid.

## Experimental

### General methods

All the compounds synthesized were characterized by NMR, IR and MS spectra. ^1^H NMR & ^13^C NMR spectra were recorded on a Bruker Avance 300 spectrometer (300 MHz). Electrospray (ES^+^) mass spectra were acquired on an ion trap mass spectrometer.

### Preparation of imidazol-1-yl-acetic acid *tert*-butyl ester (**2**)

To a solution of imidazole (10.0 g, 0.15 mol) in ethyl acetate (160 mL) was added powdered K_2_CO_3_ (29.0 g, 0.21 mol) followed by *tert*-butyl chloroacetate (25.7 mL, 0.18 mol) at room temperature and the mixture was refluxed for 10.0 h. After completion of the reaction as indicated by TLC (10% MeOH/CHCl_3_, I_2_ active), the reaction mass was quenched with cold water (80 mL) and the ethyl acetate layer was separated. The aqueous layer was extracted with ethyl acetate (2 × 80 mL) and the combined ethyl acetate layers were washed with brine, dried with anhydrous sodium sulfate and then concentrated under vacuum. The resulting solid was stirred with hexane (50 mL) at RT, filtered and washed with hexane (2 × 20 mL) to afford the title compound as an off-white solid (20.0 g, 75%). mp: 111.3–113.2 °C (Lit [10]: 111–113 °C). IR (cm^−1^): 3458, 3132, 3115, 2999, 2981, 2884, 1740, 1508, 1380, 1288, 1236, 1154, 1079, 908, 855, 819, 745, 662, 583; ^1^H NMR (300 MHz, CDCl_3_) δ 1.47 (s, 9H), 4.58 (s, 2H), 6.94 (s, 1H), 7.09 (s, 1H), 7.49 (s, 1H); ^13^C NMR (75 MHz, CDCl_3_) δ 27.7, 48.6, 82.9, 119.8, 129.2, 137.7, 166.3; MS (*m/z*) 183.0 [M+1, 100%], 127.0.

### Preparation of imidazol-1-yl-acetic acid hydrochloride (**6**)

To a solution of imidazol-1-yl-acetic acid *tert*-butyl ester (**2**) (10.0 g, 0.05 mol) in dichloromethane (100 mL) was added titanium tetrachloride (8.0 mL, 0.07 mol) dropwise slowly at −15 to −10 °C over 1 h and the mixture was stirred at −5 to 0 °C for 2 h. Isopropyl alcohol (25 mL) was added at 0 to −10 °C over 0.5 h and the reaction mass was stirred at room temperature for 0.5 h. Additional isopropyl alcohol (125 mL) was added dropwise at room temperature over 0.5 h and the mixture was stirred for 1 h. Dichloromethane was distilled out under a low vacuum and the resulting crystalline solid precipitated was filtered to afford the title compound as an off-white crystalline solid (7.4 g, 83%). mp 200.3–202.3 °C; IR (cm^−1^): 3175, 3125, 3064, 2945, 2869, 2524, 2510, 1732, 1581, 1547, 1403, 1223, 1193, 1081, 780, 650; ^1^H NMR (300 MHz, D_2_O + 3-(trimethylsilyl)propionic acid sodium salt) δ 5.1 (s, 3H, -C*H*_2_- + *H*Cl), 7.5 (br s, 2H), 8.7 (s, 1H); ^13^C NMR (75 MHz, D_2_O + 3-(trimethylsilyl)propionic acid sodium salt) 52.7, 122.4, 125.9, 138.8, 172.8; MS (*m/z*) 127.0 [M+1, 100%]; HCl-content: found 21.8% (along with 3.25% moisture), calcd 22.43% for C_5_H_6_N_2_O_2_·HCl.

### Preparation of zoledronic acid (**7**)

To a suspension of imidazol-1-yl-acetic acid hydrochloride (**6**) (7.0 g, 0.043 mol) and phosphorous acid (9.5 g, 0.116 mol) in chlorobenzene (50 mL) was added phosphorous oxychloride (9.6 ml, 0.103 mol) at 80–85 °C over a period of 2 h then heated to 90–95 °C for 2.5 h. The reaction mass was cooled to 60–65 °C and water (100 mL) was added at the same temperature. The aqueous layer was separated, collected and refluxed for 18 h. It was then cooled to room temperature and diluted with methanol (140 mL). The mixture was cooled to 0–5 °C and stirred for 3 h. The precipitated solid was filtered, washed with cold water followed by methanol and then dried under vacuum at 60 °C for 12 h to afford the title compound (6.6 g, 57% yield) as a white solid; mp 237–239 °C (lit [1] 239 °C with decomposition).

## Supporting Information

File 1 ^1^H and ^13^C NMR spectra of compound **2** and **6**.
