# Supplementary material for: Synthesis of imidazol-1-yl-acetic acid hydrochloride: A key intermediate for zoledronic acid
Source: Beilstein J Org Chem. 2008 Nov 17;4:42. doi: 10.3762/bjoc.4.42 (PMC2605618; doi:10.3762/bjoc.4.42)
Supplement: File 1 — 1H and 13C NMR spectra of compound 2 and 6. [file Beilstein_J_Org_Chem-04-42-s001.doc]

Synthesis of imidazol-1-yl-acetic acid hydrochloride: A key intermediate for zoledronic acid

Santosh Kumar Singh,* Narendra Manne, Purna Chandra Ray, Manojit Pal*

Address: New Drug Discovery, Matrix Laboratories Limited, Anrich Industrial Estate, Bollaram, Jinnaram Mandal, Medak District, Andhra Pradesh, India-502 325.

Email: Santosh Kumar Singh - [sksingh_p2@rediffmail.com](mailto:sksingh_p2@rediffmail.com); Manojit Pal* - [manojitpal@rediffmail.com](mailto:manojitpal@rediffmail.com)

*Corresponding author

**Supporting Information File 1**

^1^H and ^13^C NMR spectra of compound **2** and compound **6**.


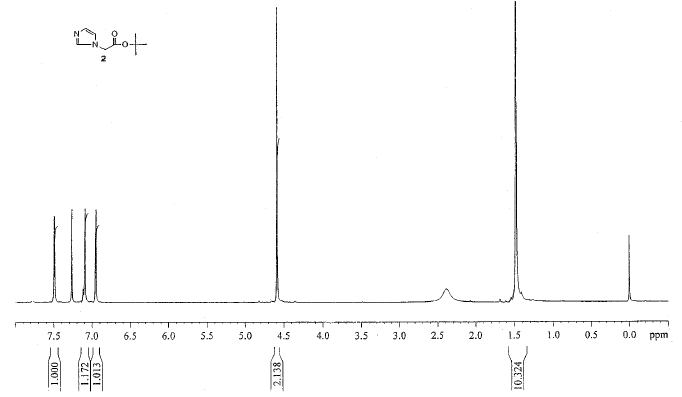


**Figure 1:** ^1^H NMR spectra of compound **2** in CDCl_3_.


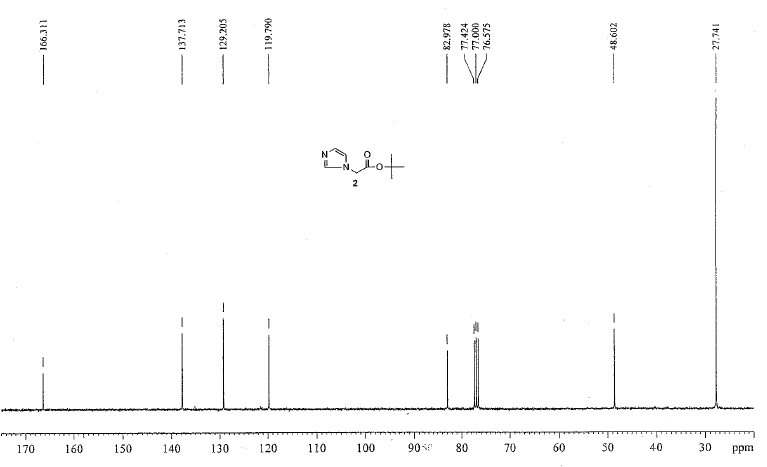


**Figure 2:** ^13^C NMR spectra of compound **2** in CDCl_3_.


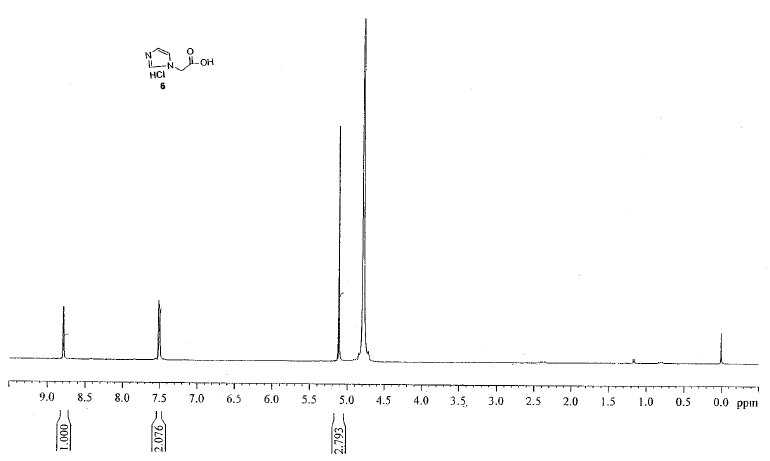


**Figure 3:** H NMR spectra of compound **6** in D_2_O + 3-(trimethylsilyl)propionic acid sodium salt.


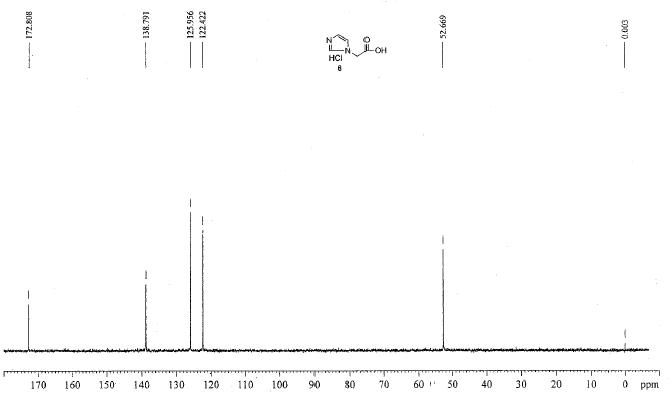


**Figure 4:** ^13^C NMR spectra of compound **6** in D_2_O + 3-(trimethylsilyl)propionic acid sodium salt.
